# Convertible and Constrained Nucleotides: The 2’-Deoxyribose 5’-C-Functionalization Approach, a French Touch

**DOI:** 10.3390/molecules26195925

**Published:** 2021-09-30

**Authors:** Crystalle Chardet, Corinne Payrastre, Béatrice Gerland, Jean-Marc Escudier

**Affiliations:** Laboratoire de Synthèse et Physico-Chimie de Molécules d′Intérêt Biologique, UMR CNRS 5068, Université Paul Sabatier, 118 Route de Narbonne, CEDEX 9, 31062 Toulouse, France; chardet@chimie.ups-tlse.fr (C.C.); payrastr@chimie.ups-tlse.fr (C.P.)

**Keywords:** oligonucleotides conjugation, convertible approach, constrained nucleic acids, convertible and constrained nucleic acids

## Abstract

Many strategies have been developed to modulate the biological or biotechnical properties of oligonucleotides by introducing new chemical functionalities or by enhancing their affinity and specificity while restricting their conformational space. Among them, we review our approach consisting of modifications of the 5’-C-position of the nucleoside sugar. This allows the introduction of an additional chemical handle at any position on the nucleotide chain without disturbing the Watson–Crick base-pairing. We show that 5’-C bromo or propargyl convertible nucleotides (CvN) are accessible in pure diastereoisomeric form, either for nucleophilic displacement or for CuAAC conjugation. Alternatively, the 5’-carbon can be connected in a stereo-controlled manner to the phosphate moiety of the nucleotide chain to generate conformationally constrained nucleotides (CNA). These allow the precise control of the sugar/phosphate backbone torsional angles. The consequent modulation of the nucleic acid shape induces outstanding stabilization properties of duplex or hairpin structures in accordance with the preorganization concept. Some biological applications of these distorted oligonucleotides are also described. Effectively, the convertible and the constrained approaches have been merged to create constrained and convertible nucleotides (C_2_NA) providing unique tools to functionalize and stabilize nucleic acids.

## 1. Introduction

Since nucleic acids were first envisaged as a serious therapeutic option and as powerful tools for biotechnical applications, their limited biostability and chemical repertoire has been constantly enhanced by chemists. The first improvements arose by conjugating additional functionalities depending on the desired applications while sharing the converging purpose of increasing their in vivo properties, as well-reviewed by Ochoa and Milam. [1] Structural modifications were proposed to increase their affinity, specificity, and enzymatic and chemical stability [2]. We focus here on the recent development of oligonucleotide (ON) conjugation while describing our contribution to the field which is aimed at the development of chemical modification at the 5’-C-position of the sugar moiety of the nucleotide leading to 5’-C-introduction of a chemical handle, or a label of interest, by means of a convertible or functionalized approach, respectively. This chemistry has allowed us, in an alternative manner, to describe the use and impact of structural constraints on oligonucleotides by covalently restricting the conformation of torsional angles, based on the introduction of a 1,3,2,-dioxaphosphorinane ring within the sugar-phosphate backbone. Ultimately, we demonstrated that the two strategies, both functionalization and structuration, could be applied in a synergistic way to functional oligonucleotides.

Oligonucleotides can be modified covalently to increase their resistance to nucleases to survive in vivo antisens therapy [3], as well as to enhance their cellular uptake or targeting efficiency, by grafting onto them either fatty acids [4], peptides [5,6], or small molecules, such as the GalNAc modification [7]. These modifications are also of particular relevance in biosensing [8], in imaging [9] and cancer theranostics [10], and in the ever-growing fields of DNA-hybrid catalysis [11,12] and DNA origami technology [13].

**Scheme 1:**
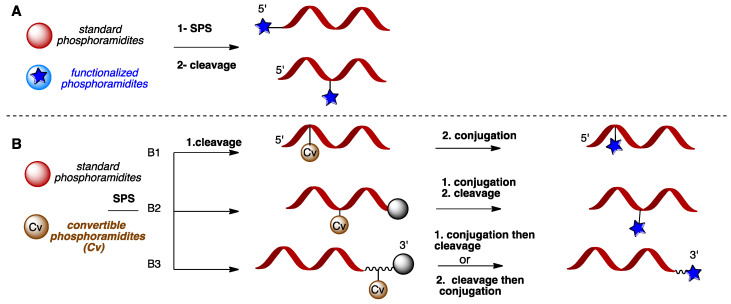
Schematic representation of the two strategies (illustrated with the supported phosphoramidite synthesis) used for the synthesis of functionalized ONs either by: (**A**) incorporation of an already functionalized building block; (**B**) incorporation of the convertible handle by SPS, followed by conjugation in solution (B1), or on-support conjugation, followed by deprotection and cleavage (B2), and incorporation at the 3’-position of the oligonucleotide (B3) with conjugation either before or after cleavage from the support.

However, even if all these applications share a biological purpose, they all rely on chemical means. In the first instance, efficient oligonucleotide synthesis is no longer an issue as a result of the development of reliable DNA solid-phase synthesis (SPS) based on phosphoramidite chemistry. Secondly, the use of handles to introduce modifications onto oligonucleotides is well-documented since the 1990s in the nucleic acid literature [14] and was already described in 1994 by Verdine [15] who coined the term ‘convertible approach’, [16] and was reviewed more recently by Defrancq’s team [17]. However, with the same final goal of conjugation, the timeline for the introduction of the modification requires almost two opposite strategies. One is based on the incorporation of a monomer (phosphoramidite or a *H*-phosphonate building block), already decorated with the suitably protected conjugate, leading after the final (harsh) ammoniac deprotection to the functional oligonucleotide (Scheme 1, strategy A). The main hurdle is the time-consuming, and sometimes challenging, chemical synthesis of the required building blocks, as the expressed reactive functionalities are required to be compatible (or at least the protected groups have to be) with all SPS cycles. In this respect, strategy B relies on a late-stage functionalization of an oligonucleotide by incorporation of a convertible handle, inert to the SPS conditions, with the advantage of being either commercially available or easily synthetized. Post-synthetic coupling reactions circumvent the issues of incompatible chemistry between ONs and the desired conjugated species, since they are synthetized apart. However, the reaction has to be very efficient at low concentrations and to be DNA and water-compatible or, at least, an organic miscible solvent should be employed. Strategy B is also the preferred option to obtain conjugated ONs by enzymatic polymerization of nucleoside triphosphates, as smaller convertible modifications are better tolerated by the engineered polymerases in control [18,19].

In both cases, conjugation can be performed either at the 3’- or 5’- end of the oligonucleotide or at an internal position, the two latter being the preferred options when incorporating already functionalized phosphoramidites decorated with the molecule of interest (Scheme 1, strategy A). Building blocks usually derive from modified nucleotides, but not always [20], and handles are usually located on the bases at positions that are first easily accessed by chemistry, such as the C8 position on purine (mainly adenine), or on the extensively studied C5 position on pyrimidine (with a preference for deoxyuridine derivatives). The Watson–Crick base-pairing would retain its structural integrity in those examples with a major groove orientation of the handle within a duplex. The 2’-position on the sugar could also be a site of labeling, when a minor groove orientation is desired, for minimal disruption of the duplex. It is also of interest to perform the conjugation on-support (Scheme 1, strategy B2) before the ammonia treatment, as a slight excess of the conjugate partner is sometimes required to ensure the completion of the coupling. While the ON is still covalently linked, the washing step is implemented easily and proven to be more effective to remove and recover (if necessary) the unreacted material [21]. Finally, modifications at the 3’-end (strategy B3) are made possible by the chemical modification of the support, and conjugation could be performed either on-support or in-solution [22].

## 2. Chemical Strategies for Post-Synthetic Functionalization

**Scheme 2:**
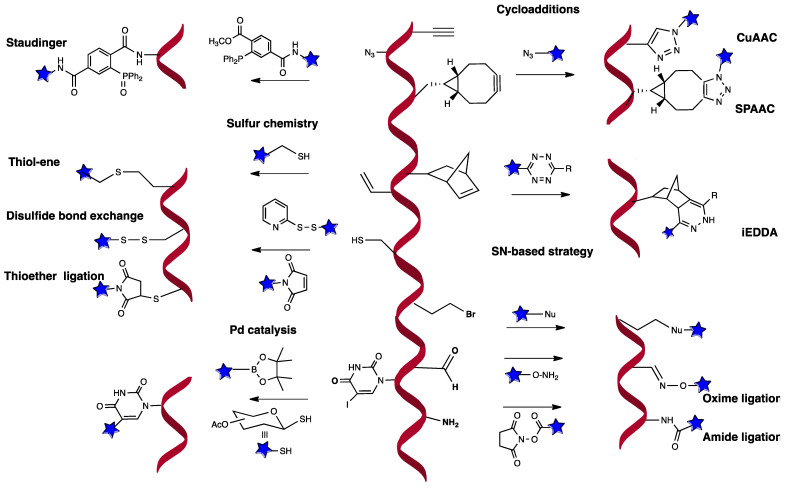
Post-synthesis labeling strategies onto ONs.

The part of this review devoted to the functionalization of ONs will be a non-exhaustive overview of the chemical handles used for the convertible approach in the last few years, as this appears to be a combination of well-established conjugation reactions and emerging labeling methods (Scheme 2).

### 2.1. Nucleophilic Displacement

Depending on the application, linkages are designed to be either cleavable or stable. Numerous commercially convertible phosphoramidites are available and mostly present either the popular amine or thiol group onto the building blocks or the support. Phosphoramidites, bearing a masked amino group, are of particular interest to obtain post-SPS 5’-amino modified oligonucleotides, as they provide an anchor for the introduction of organic molecules during the DNA-directed template synthesis [23]. Indeed, the amide bond formation is one of the most used reactions to selectively obtain macrocycle libraries due to the unsurpassed ability of DNA to bring, in a programmed manner, two reactants together by increasing the effective concentration through the hybridization process [24]. The Roelfes’ team developed a modular assembly of functionalized oligonucleotides with terminal amino linkers reacting with an NHS-activated bipyridine species to explore the impact of the covalently linked ligand on the enantioselectivity of C-C bond formation reactions [25]. A recent addition to the toolbox of bio-orthogonal ligations is the sulfur-fluoride exchange (SuFEx) technology, based on the reaction between a thionyl tetrafluoride(SOF_4_)-based partner and a 5’-amino-ON to form a sulfamide-labeled ON [26].

Another efficient ligation is based on the straightforward nucleophilic substitution of an electrophilic handle. This ligation usually has to be performed on a support to prevent ammonia displacement but presents the advantage of an easy removal/recovery of the unreacted, unbound reactants while maintaining the ON on the resin. The nature of the electrophilic group could be a halide (bromine) atom [27] or a methyl ester [28]. An elegant approach was developed while studying the DNA-catalyzed allylic amination using a linked diene-iridium (I) ligand obtained by nucleophilic substitution of the 4-triazolyl-desoxyuridine [29].

Numerous efficient conjugations are built on the reactivity of amino-oxy functions with aldehydes to form an oxyme bond stable in the physiological pH range, in a chemoselective and efficient fashion. Being best performed at slightly acidic pH, the first example of convertible ON was obtained post-synthetically after oxidation of a residual glycerol group to an aldehyde later engaged with aminooxy substrates [30]. An alternative was the use of masked aminooxy groups as phthaloyl groups, liberated after SPS by the action of hydrazinium salts [31,32]. Recent examples have demonstrated the added value of the presence of the aminooxy function at the 3’-end to modulate the length of the spacer [33].

Michael-type addition between thiols and maleimides is another popular solution to covalently label ON in a selective fashion when performed at neutral pH. Depending on the strategy, either the protected forms of maleimide [34], or the thiol handle [35], could present on the ON, but they have to be liberated after SPS by ammonia treatment or in reducing conditions, respectively, to react with their counterparts. Starting from a free thiol function on the ON, the partners could be linked through a disulfide bridge after a disulfide bond exchange, usually with a pyridyldithiol-activated species [36]. This approach was recently reviewed [37], mostly to study transient non-canonical DNA secondary structures. One key feature (for better or for worse) is also the reversibility of such a bond, cleaved under reducing conditions [38]. Thiol-ene chemistry was also exemplified by the Hocek group, reaching good coupling yield without the need of UV irradiation [39,40].

### 2.2. Cycloadditions

Unlike the amine or thiol functional groups, that have to be protected to undergo the SPS cycles before deblocking, the alkyne group is, in principle, chemically resistant to all the steps [41]. Indeed, one of, if not the most used conjugation reaction is the Cu(I)-catalyzed alkyne-azide 1,3-dipolar cycloaddition (CuAAC) [42] due to its bio-orthogonality and its chemical efficiency, and is encompassed in the ‘click chemistry’ reactions field. Moreover, it is so commonly used that it is sometimes incorrectly referred as the sole ‘click’ reaction. In addition, the resulting 1,2,3-triazole ring is considered to be inert in vivo but could also act as a bio-isoster of the amide bond. Starting from the 5-ethynyl- or octadiynyl-dU derivatives [43,44], recent improvements were directed towards the synthesis of 5-dU phosphoramidites bearing longer and flexible linkers [45]. Following the development of DNA-encoded libraries, the introduction of aryl amines onto a duplex bearing a terminal alkyne was realized by a one-pot efficient reaction by in situ generation of aryl azides from TMS-N_3_ and aryl borate derivatives in the presence of a Cu^2+^/β-cyclodextrine complex [46]. The CuAAC reaction could even be optimized to be performed during the supported automated synthesis with an on-synthesizer protocol using CuI·P(OEt)_3_/DMAA reagents [47]. While the consumption of reagents is minimal and the gain of time optimal, the main drawback resides in the mandatory stability of the conjugate during the ammoniac final deprotection. To lower the quantity of toxic copper, different strategies have been employed, including the use of chemically designed azide-chelating species, or by relying on a biological source of sequestered copper within the human copper(I)-binding chaperon Cox17, mediating an efficient labeling of alkyne-modified ON [48]. 

Finally, the development of the SPAAC (strain-promoted azide-alkyne cycloaddition) as a copper-free conjugation alternative was illustrated by the introduction, during the standard SPS, of the bicyclo[6.1.0](non-4-yn-9-yl)ethan-1-ol (BCN) phosphoramidite at the terminal 5’-position, followed by reaction with chondroitin sulfate azide precursors for application for the DNA-directed immobilization (DDI) of carbohydrates [49]. Although aiming at in vivo applications circumventing the toxicity of copper ions, the downside of this approach is the slower reaction rate compared to the classic CuAAC reaction. On the other hand, the presence of an azido group on the ON appears to be more problematic. It was generally accepted that the Staudinger reaction between the P(III) atom of any phosphoramidite and an azido organic function was detrimental to the formation of azide conjugated ON by SPS [50]. In order to by-pass this issue, it usually requires a time-consuming late-stage introduction of the azido handle. For example, an oligonucleotide bearing a terminal amino linker underwent a peptide coupling first with a corresponding glycine as a proof of concept, then with a variety of amino-acids or alkyle derivatives. The resulting free amino group was later converted into an azide group through a diazo transfer with the popular and shelf-stable imidazole-1-sulfonyl azide salt [51]. A Staudinger ligation was nicely designed onto a post-synthesis azide-labeled ODN to introduce a fluorescent dye bearing a triphenylphosphine group [52].

However, examples indicated that the difference of reaction rates between the tetrazole-catalyzed coupling and Staudinger degradation did not impair the formation of a growing ON immobilized onto an azide decorated support [53], but that the use of a phosphoramidite bearing an azide group is possible at the cost of very careful storage [54].

Orthogonal to the CuAAC bioconjugation process, the Diels–Alder conjugation was described between dienes-modified ON (hexadiene [55] or furan [56] phosphoramidites) and maleimides substrates. Then, displaying more impressive kinetic rates is, firstly, the inverse-electron-demand Diels–Alder cycloaddition (iEDDA) with the site-specific incorporation of norbonene-derived phosphoramidites [57]. This could react with tetrazine partners as electron-poor dienes, and secondly, its strain-promoted version (SPIEDAC) using the *trans*-cyclooctene (TCO) [58] and BCN modified-phosphoramidites bearing the dienophile function. Despite displaying both efficiency in mild conditions with N_2_ as the only inert by-product, unfortunately concerns arose as some iEDDA components were revealed to be unstable, either to the repetitive use of iodine during the iterative SPS, or to the deprotection conditions, requiring the tetrazine partner to be introduced post-synthetically. Optimizations were achieved by switching to 1,2,4-triazine-modified nucleoside tri-phosphates as diene partners to increase the overall stability [59]. It is also worth mentioning that the photo-click methodology relies on a photochemical process as a bio-orthogonal metal-free option [60,61]. The induced cycloaddition by a selective, but not detrimental, LED irradiation at 365 nm, between photoactivable diaryltetrazoles handles introduced onto ODN during SPS, and activated alkenes (such as maleimide derivatives) produced an under-rated alternative, in particular for metabolic labeling [62].

### 2.3. Palladium Catalyzed Conjugation

The powerful Pd-catalyzed Suzuki–Miyaura cross-coupling reaction also relies on the presence of a halide atom (either bromide or iodide) to label ODN through C-C bonds a post-synthetically via C^8^-aryl purine [63] or C^5^-uracile [64] adducts. It should be emphasized that this reaction is totally bio-orthogonal, performed in water in mild conditions. This expanding field also explored the Sonogashira and the Stille–Migita reactions, their scope and limitations having been recently reviewed [65]. Although mostly limited to the introduction of small molecules, the Pd-catalyzed conjugation was demonstrated with the selective conjugation of the Z33-N17C protein at the 5’-amino terminal position of ON, through the formation of stable ON-Pd(II) oxidative addition complexes as intermediates [66]. Buchwald–Hartwig–Migita cross-coupling was also described to occur on a 5-iodo-uracile modification incorporating phosphoramidite chemistry to lead to thioglycosylated oligonucleotides [67].

The key feature of all of these developed conjugation reactions is that they are usually orthogonal to each other, allowing the labeling of at least two different molecules. The CuAAC click reaction is usually involved, either by modulating the reactivity of the ethynyl groups in a sequential fashion [68], or by being combined either with an iEDDA reaction [69], a nucleophilic substitution [28], an oxime ligation [33,70], or a thiol-Michael addition [71].

### 2.4. Narrowing to the 5’-C Functionalization of Nucleic Acids

However, as biological chemists we were interested in a long-term program of research devoted to ON conjugation and shape control. We therefore decided to take advantage of the expanding bio-orthogonal labeling toolbox to exemplify 5’-C-modifications at this position. Examples were scarce, despite allowing the introduction at any position along the ON backbone without the need of an abasic site. The chemistry of this 5’-carbon atom inevitably produces diastereoisomers, unless either excellent stereoselectivity is reached or analytical separation conditions are found. Moreover, we anticipated that the increased steric hindrance of an additional chemical handle at this position would not impair the coupling yield during the SPS.

**Scheme 3:**
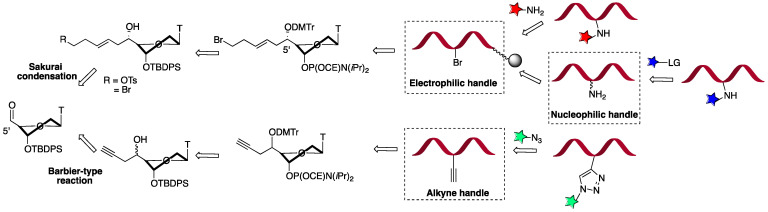
Retrosynthesis analysis of convertible nucleotide (CvN) and bromine or alkyne functionalized ON from thymidine aldehyde.

We then embarked on a long journey to illustrate conjugation at the 5’-C-position, mostly based on two major convertible nucleotides (CvN). One bears a bromide atom on a pentenyl arm as a leaving group that can be displaced easily, either directly by the desired nucleophilic (amino) conjugate, or by a bi-nucleophilic species (such as a diamino-alkane type) molecule that inverts the handle to a nucleophile. The second convertible was designed to present an alkyne function for CuAAC reactions (Scheme 3).

#### 2.4.1. 5’-C-Bromo or Tosyl Pentenyl Thymidine as Convertible Nucleotides (CvN)

Synthesis of nucleotides was relatively straightforward and originated from a common 3’-protected-5’-aldehyde thymidine.

For (*S*)-5’-C-(5-bromo-2-penten-1-yl)-2’-deoxyribofuranosyl thymine phosphoramidites, the first step was a Sakuraï condensation leading to the formation of a tetrahydrofuran ring with a different diastereoselective outcome depending on the nature of the corresponding 5-substituted-3-trimethylsilylpentene [27,72]. After the ring opening in the presence of titanium tetrachloride and a protection-deprotection sequence, the (*S*)-5’-C-(5-bromo-2-penten-1-yl)-2’-deoxyribofuranosyl thymine was phosphitylated to give access to the bromo-convertible phosphoramidite.

The synthesis of a 5’-C-alkenyle nucleotide was realized in one step by reacting the previously described 5’-C-aldehyde thymidine with propargyl bromide in a Barbier-type reaction, leading to a mixture of diastereoisomers in a *S/R* = 70:30 ratio. Although both nucleosides could be separated by direct phase HPLC, they could be engaged as a mixture to give, after 5’-*O*-protection with a *p*-dimethoxytrityl group (DMTr), a 3’-*O*-déprotection by fluoride ions and, through a final 3’-*O*-phosphitylation procedure, the corresponding alkyne-convertible phosphoramidites [73].

**Scheme 4:**
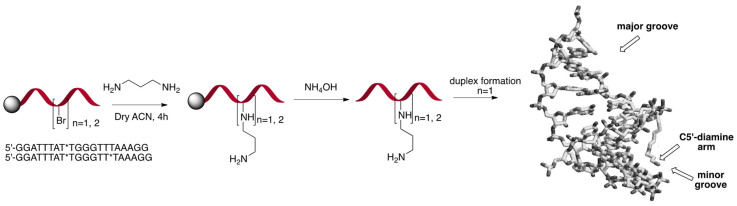
Bromine displacement by diamine and molecular modeling of the resulting duplex, T* = 5’-C-bromopentenyl thymidine.

Firstly, the 5’-C-bromopentenyl-phosphoramidite CvN was smoothly incorporated during the SPS iterative process at the terminal 5’-position with the same efficiency as the commercial non-modified phosphoramidites. The oligonucleotide was deprotected and cleaved from the support by an ammonia treatment that consequently displaced the electrophilic bromine atom in the process. The newly available 5’-amino group present on the ON reacted selectively as the only nucleophilic entity with a rhodamine activated ester, as a proof of concept. Secondly, the impact of the (*S*) stereochemistry of the C5’ atom was evaluated on an ON modified at internal positions by substitution with propane-1,3-diamine performed on-support followed by ammonia cleavage and deprotection. A computational model highlighted the minor groove orientation of the handle within an RNA/DNA duplex (Scheme 4). The introduction of the newly generated stereogenic C5’ center then allows the possibility to direct modifications toward a different environment, as a 120° deviation in the orientation of the residue between the C5’-(*S*) and C5’-(*R*) is observed within a duplex.

New applications were subsequently envisaged, in particular the ON conjugation to macrocyclic lanthanide complexes [74]. At that time, the purpose was to further explore the properties of the europium lanthanide ions chelated by a neutral tetraazamacrocycle ligand (cyclen) in the context of their covalent attachment to ON, and to study their potential catalytic properties towards non-enzymatic phosphodiester cleavage under physiological conditions [75].

**Scheme 5:**
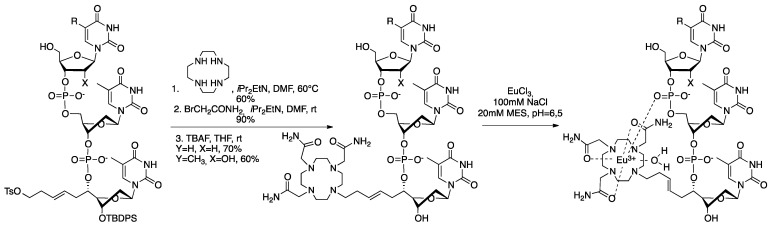
Synthesis of Eu^3+^ cyclen complex conjugated to trinucleotides as a nuclease mimic.

The in-solution synthesis of two trinucleotides (in deoxy and ribo series) bearing a tosylate-convertible nucleoside at the 3’-end was proven to be without event, following the same synthetic sequence as the one described to obtain its bromo counterparts. Starting from the original thymidine 5’-aldehyde reacting with ω-tosyloxy substituted allyltrimethylsilane, the resulting CvN (5*S*)-5’-C-(5-tosyl-2-penten-1-yl)-2’-deoxy ribofuranosyl thymidine, bearing the tosyl group as a leaving group, was coupled using a phosphoramidite chemistry-based protocol. The resulting dinucleotide was then again coupled, either with a commercial thymidine or uridine phoshoramidite. A triethylamine treatment selectively removed the cyanoethyl phosphate protecting group to give 5’-TTT* or 5’-UTT* trinucleotides as triethylammonium salts bearing the tosyl handle at the terminal 3’ position. Although the introduction of the (1,4,7-tris (carbamoylmethyl)-1,4,7,10-tetraazacyclododecane) presenting the three pendant amide groups was unsuccessful, the lack of reactivity of the remaining amine was by-passed by the introduction of the corresponding unsubstituted cyclen. The remaining amino functions were later selectively alkylated by bromoacetamide before the last deprotection step involving the use of fluoride ions to liberate the terminal 3’-hydroxyl function (Scheme 5).

Upon complexion of EuCl_3_ with the two free trinucleotides, luminescence studies indicated that both Eu(III)-nucleotide complexes were formed in water, corroborated by the presence of a major peak at 579.5 nm, and at 579.6 nm for the DNA-only or with chimeric RNA/DNA Eu(III) conjugates. Moreover, a water molecule and one of the phosphate esters acting as an intramolecular ninth donor group were described to complete the coordination sphere of Eu(III). Finally, cleavage of the phosphodiester bond, due to the hard Lewis acid properties of the lanthanide ion acting as a metal co-factor, was corroborated by the detection of the corresponding uridine 2’,3’-cP, however with a modest rate constant of 1.3 × 10^−5^ s^−1^.

**Figure 1:**
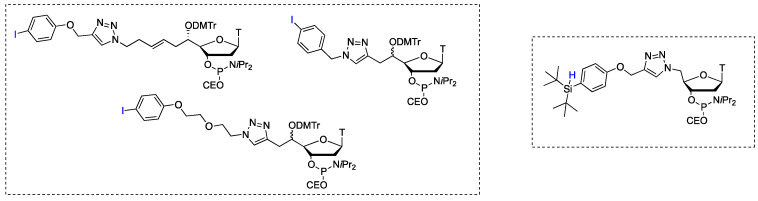
Already functionalized phosphoramidites for ^11^C (**left**) or ^18^F (**right**) labeling application.

Our team was also involved in developing modified nucleotides and oligonucleotides to allow the site-specific, late-stage introduction of short half-life radioisotopes such as ^11^C (t_1/2_ = 20.4 min) and ^18^F (t_1/2_ = 109.6 min) for positron emission tomography (PET) applications. Such conjugation must achieve challenging outcomes, such as chemical compatibility between partners, and one-step coupling efficiency in aqueous and diluted conditions. The palladium-catalyzed Stille reaction had been previously developed to realize a ^11^C-methyl transfer reaction based on ^11^C-labeled hypervalent methylstannate originated from ^11^C-methyl iodide onto an iodo-arylpartner [76]. For ease of access, the synthesis of already functionalized phosphoramidites bearing the iodoaryl moiety was designed based on the CuAAC reaction, implying that either an azide or an alkyne function had to be present on the convertible nucleoside (Figure 1). For the former option, (5*S*)-5’-C-(5-bromo-2-penten-1-yl)-2’-deoxyribofuranosyl thymidine was converted into its azido counterparts to further react with the iodoaryl alkenyl derivative [73].

The methyl transfer protocol optimized for small organic molecules had to be adapted for biomolecules to overcome inherent issues, such as the necessity to work in more dilute conditions, and using a mixture of solvents, as ONs are not soluble in DMF-only solutions. One key feature to study was also the impact of the phosphodiester linkage. As the reaction requires high temperatures detrimental to the stability of ONs, microwave activation was used without the presence of copper as a first proof of concept on a dinucleotide. The palladium-catalyzed reaction went to completion within 5 min with no detected formation of the hydrogenated by-product [77].

For the design of new PET radiotracers, the introduction of ^18^F in a late stage and in a one-step labeling fashion is still challenging, and the usual methodology requires harsh (basic and high temperatures) and dry conditions that are not compatible with ONs [78].

**Scheme 6:**
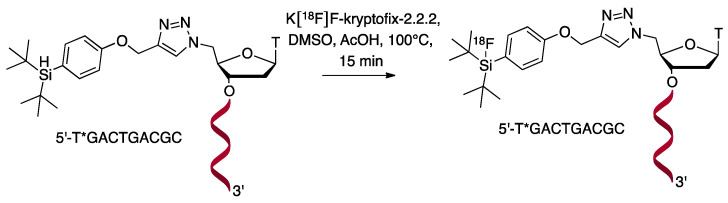
^18^F labeling of ON.

While progress has been made since [79,80], examples of direct ^18^F fluoration of oligonucleotides and aptamers were rare at the time [81], especially when based on silicon fluoride chemistry. An already functionalized phosphoramidite, bearing at its 5’-C position a di-tert-butylsilyl group known to stabilize the Si-F bond in the most efficient way in physiological conditions, was introduced into a 10mer ON at the 5’-end. The best conditions developed for a direct ^18^F fluoration onto an ON were optimized in 36% not-decay-corrected yield with a 95% radiochemical purity (Scheme 6). No detrimental effect on the stability (in particular no depurination) was observed and the presence of numerous phosphodiester bonds was proven to be compatible with this silicon-fluoride chemistry.

A non-metal-based application was also developed through an elegant fluorogenic approach. Incorporation of phosphoramidites, already bearing the fluorescent handle, could lead to degradation due to either the acidic or basic steps during SPS, and requires additional synthetic steps to obtain a suitably protected dye. On the other hand, conjugation post-SPS is generally performed with an excess of the fluorescent partner, making somewhat tedious the thorough washing of the unreacted material.

**Scheme 7:**
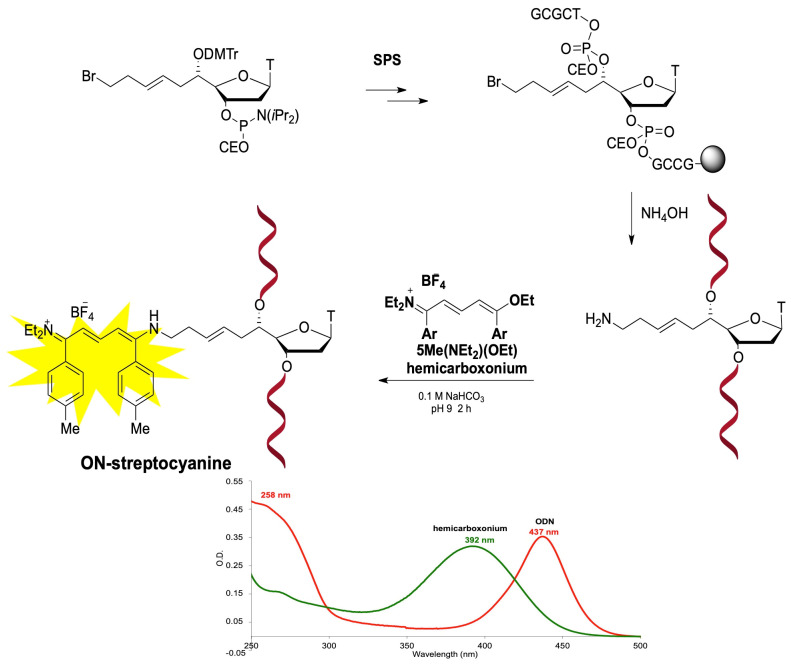
Fluorogenic labeling methodology of ON with hemicarboxonium salt and UV spectra of hemicarboxonium (green) and of streptocyanine-labeled ON (red) in pH 7 phosphate buffer.

In that context, designing two non-fluorescent partners that would exhibit fluorescence only when conjugated in a fluorogenic approach would circumvent the two former issues. Until then, the main strategy was based mostly on the modulation of the photophysical properties of coumarin derivatives that remain non-fluorescent until the CuAAC reaction results in the formation of the fluorescent product [82,83]. One of the key issues was also to take advantage of a direct fluorescent readout confirming that the two partners were in close proximity throughout the increase of the effective molarity with application in the DNA-templated detection or ligation. Bearing this in mind, we developed our own fluorogenic approach based on a nucleophilic substitution of an ethoxy group present on the non-fluorescent hemicarboxonium salts by a primary amine-modified ON, leading to a highly fluorescent streptocyanine conjugate. The 10-mer ON bearing an internal amino handle was obtained from the convertible bromo alkyl-ON, as previously described, and then reacted in aqueous buffered conditions with the corresponding hemicarboxonium salts to yield a streptocyanine-conjugated ON (Scheme 7). Completion of the conjugation reaction could be nicely demonstrated by the analysis of the UV spectrum solely by the presence of both the 258 nm band, characteristic for nucleic acids, and an additional 437 nm band, a signature of the streptocyanine dye.

It is worth mentioning the observed chemo-selectivity as none of the exocyclic amines from the nucleobases reacted, despite a 20-fold excess of hemicarboxonium salts. Denaturation studies indicated that the introduction of the fluorogenic species was only slightly destabilizing in both DNA duplexes by around −1 °C. In addition, no bleaching was observed during these experiments. In the context of single nucleotide polymorphism (SNP) applications, the ability of these streptocyanine-conjugated ONs to discriminate the presence of mismatches within DNA or DNA/RNA duplexes was evaluated by monitoring the changes in the fluorescence data. However, a modest fluorescence enhancement was observed only when a single mismatch was present in the studied DNA/RNA duplexes, highlighting the impact of the orientation of the dye on the fluorescence intensity.

#### 2.4.2. 5’-C-propargyl Thymidine as Convertible Nucleotide for the Construction of Protease Mimics

It should be noted that most of the literature on DNA-based catalysis has revolved around oligonucleotides bearing metallic co-factors, such as the very elegant approach proposed by Willner’s laboratory based on a new family of ‘nucleoaptazymes’ to mimic the cathecol oxidase activity [84]. Long-term research work from the Perrin laboratory has sought to mimic ribonucleases by cleaving the phosphodiester bond of an RNA target with sequence selectivity with a M^2+^-independent RNA-cleaving DNAzyme bearing either imidazole, cationic amines, or the guanidine group from [85].

We decided to focus on and to study the modification of oligonucleotides with amino acid-like functions to realize the hydrolysis of the amide bond by designing artificial DNA-based proteases, and we have described in this review the most recent results in this area. As protease-mimic design has been an active field for decades, the efficiency of these enzymes has not been matched yet by any synthetic scaffolds [86]. From all the designed scaffolds, the exquisite ability of DNA to (re)fold to any desired programmed secondary structure could allow the accurate positioning of the involved amino acids, in an effort to reproduce the spatial organization of the catalytic triad made of the three cooperative amino acids (Ser), (His) and (Asp). Based on a SELEX approach, the initial use of unmodified nucleoside 5’-triphosphates has met with limited success [87,88]. However, the functionalization of purine and pyrimidine 5-triphosphates [89], and the thorough work of the Silverman laboratory on modified uridine-5′-triphosphates bearing a primary amino, hydroxyl or carboxylate group, allowed the isolation of a catalytic DNAzyme working, however, with high concentrations of metal ions [90]. Relying on the solid phase bottom-up approach, Madder’s team developed 2′-functionalized uridine phosphoramidites bearing amino-acid-side-chain-like residues tethered to the oligonucleotide via an amide bond [91]. In all examples, only modifications at the 5-position on the base or at the 2′-OH were described. As the structuration properties of DNA have not been exploited to their full potential, we decided to build a 3WJ, bearing at its core the three functional groups involved in the catalytic triad [92].

**Scheme 8:**
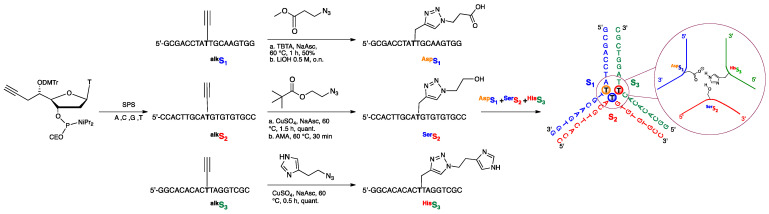
Construction of a DNA three-way junction decorated with amino acid function surrogates.

We chose to establish our proof of concept relying on a versatile convertible approach, firstly by introducing the organic functionalities at the 5’-position, and secondly by organizing our potential DNA-based proteases into a flexible secondary structure such as the 3-way junction. This secondary structure was envisaged, as its known core flexibility would allow the accommodation of a substrate in close proximity with the appended modifications. A convertible 5′-C-(*S*)-propargyl thymidine phosphoramidite was incorporated successfully into three different oligonucleotides. After supported synthesis, each strand underwent a post-synthetic CuAAC click reaction with an azide derivative bearing either a carboxylate, an alcohol, or an imidazole function surrogate for the lateral chains of aspartate, serine, and histidine respectively. The three functionalized ONs were then annealed in an equimolar fashion leading to a DNA 3WJ, bearing at its core the three unpaired modified thymidines (Scheme 8).

The assembly was confirmed by polyacrylamide gel electrophoresis, and the circular dichroism studies confirmed that each duplex arm was in its expected B-DNA form. A closer examination of the thermal denaturation data allowed us to evaluate the impact of each modification. The introduction of a carboxylate moiety was modestly destabilizing, neutral for the alcohol one, and slightly stabilizing for the imidazole residue, in accordance with their expected electrostatic interaction with the negatively charged oligonucleotides. Both the unmodified, triply propargyl- and the Ser, His, Asp modified 3-way junctions displayed the same Tm value of 43 °C, allowing us to conclude that the three incorporated modifications did not stabilize the secondary structures but had no deleterious impact on the steric hindrance of the resulting cycloaddition product. These results, taken together, demonstrated that the choice of functionalized 3WJ structure is probably the right compromise between flexibility and stability.

## 3. Structuration of Nucleic Acids (CNA)

Of course, when speaking about nucleic acid structuration, one could think about formation of 2 or 3D structures based on DNA supramolecular assembly to build outstanding architecture by the very powerful origami approach [13,93].

**Figure 2:**
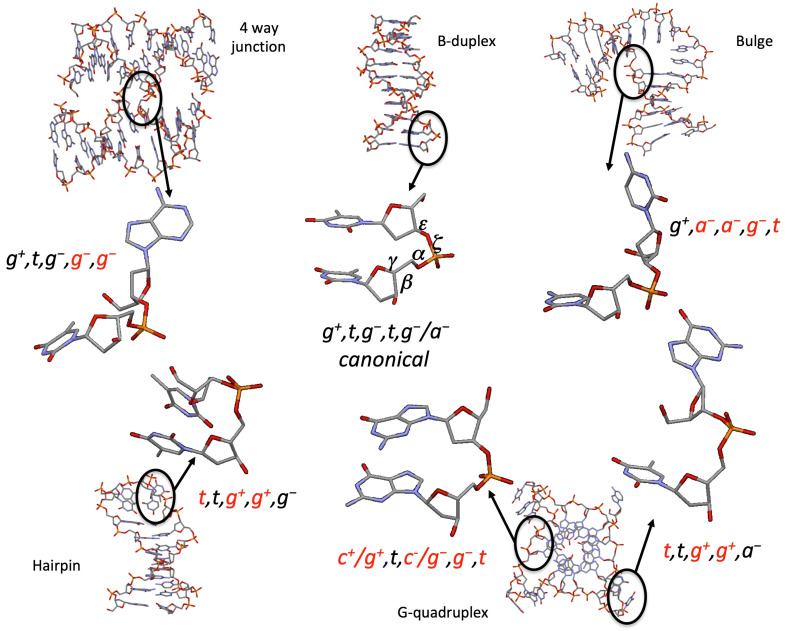
Examples of DNA secondary structures and associated backbone torsion angles γ/β/α/ε/ζ of representative dinucleotide units. The following 6-fold staggered pattern of the torsional angles is used: cis = 0 ± 30° (c), gauche(+) = 60 ± 30° (g^+^), anticlinal(+) = 120 ± 30° (a^+^), trans = 180 ± 30° (t), anticlinal(−) = 240 ± 30° (a^−^), gauche(−) = 300 ± 30° (g^−^). The notation g^−^/ a^−^ is used to designate a torsion angle on the border of gauche(−) and anticlinal(−). PDB ID: Hairpin, 1ii1; duplex, 436d; Bulge, 1jrv; 4-way junction, 1zez; G-quad, 1kf1.

As biological chemists, we have a molecular vision of the oligomer, and our vision of the structuration of nucleic acids is in the mimics design of particular structural features that can be adopted by the sugar/phosphate backbone of nucleic acids in biologically relevant secondary structures, such as duplexes, hairpins, bulges, 4-way junctions, or G-quadruplexes (Figure 2) [94].

Within a regular A- or B-duplex, each nucleotide adopts a conformation in which its sugar phosphate torsional angles are clustered in values denoted as canonical (typically: g^−^, t, g^+^, g^−^/a^−^, t, for the B-form) [95]. However, in order to be able to adopt other biologically relevant secondary structures, the sugar/phosphate conformation is distorted, and therefore, locally exhibits torsional angle values that significantly deviate from the canonical ones (Figure 2).

**Figure 3:**
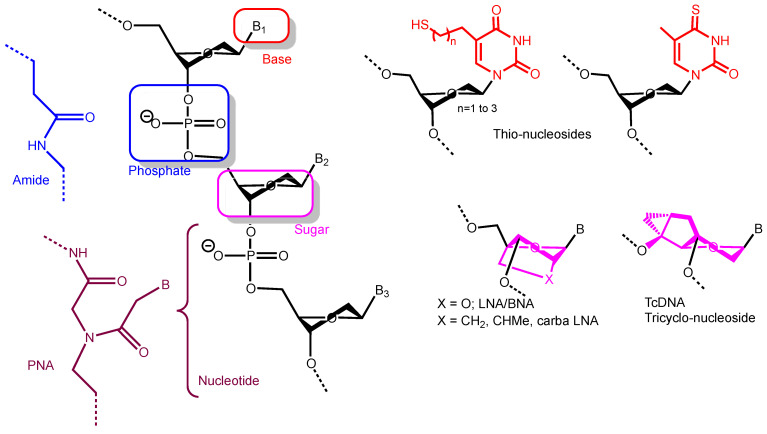
Examples of nucleoside analogues used to constrain nucleic acids structures.

Because most, if not all, of the expected applications of nucleic acid analogues rely on their ability to form stable and specific helical complexes to a target nucleic acid, much attention has been devoted to the design of synthetic analogues with enhanced binding properties, and/or enzymatic stability [96,97,98,99,100]. A conformational constraint can be applied to nucleic acids in many ways, either by base, sugar, or phosphate moiety modifications. In that context, among all the introduced modifications, a few emerged as prominent examples of a class of compounds that induced conformational restriction to nucleic acids (Figure 3) [101,102].

Thiol-modified nucleosides developed by Glick and co-workers are compounds bearing a sulphide function on the base [103,104]. They were designed to be incorporated at precise locations within each strand of DNA or RNA in order to form disulfide cross-linked structures and locked, either duplex or hairpin, structures.

Among all the sugar-puckering conformational restrictions described, the tricyclo-DNA described by Leumann’s group [105], and the LNA/BNA synthesized in the Imanishi [106] and Wengel laboratories [107], together with newly introduced carba-LNA and BNA analogues, are modifications inducing high duplex stabilization (up to +3° and +8° C/mod respectively), mainly due to the entropic benefit provided by a preorganized sugar conformation [108]. The integration of LNA moieties on every third position changes the structure of the double helix from the B to the A type. This conformation allows a much better stacking and then a higher stability. Nevertheless, so far LNA, in which only the delta torsion angle of the ribose is constrained, represents the best ribonucleoside analogue and exhibits many biological properties and applications because it can form the strongest duplex with the RNA targets [109].

On the other hand, conformational rigidity has also been introduced by replacing the internucleotidic phosphodiester moiety by amide linkages with good success [110].

Eventually, both phosphate and sugar moieties can be replaced by amide-bonding, directly connected to the base; to date the peptidic nucleic acids (PNA) represent the modification that induces the major stabilization effect in nucleic acids, mainly due to their neutrality and conformational restriction [111,112].

**Figure 4:**
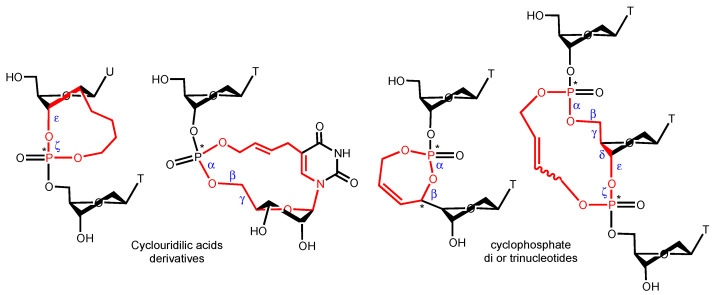
Cyclophosphotriesters obtained by RCM (* newly generated asymetric center).

Inspired by Sekine’s pioneering work on cyclic uridilic derivatives designed to bend DNA [113,114,115,116], Poul Nielsen’s group showed that the ring-closing metathesis (RCM) method was suitable for the construction of conformationally restricted dinucleotide structures (Figure 4). They developed this methodology to provide new tools to pre-organize a single stranded nucleic acid, either to form stabilized duplexes, or to induce stabilization in other secondary structures, such as three-way junctions [117,118,119,120,121,122].

However, this approach towards conformationally-constrained nucleic acids relies on the preparation of macrocyclic structures and, as a consequence, the conformations of such molecules are not well-defined being relatively flexible due to the lack of rigidity of cycles larger than a 6-member ring.

### 3.1. Dioxaphosphorinane Constrained Nucleic Acids Approach

**Figure 5:**
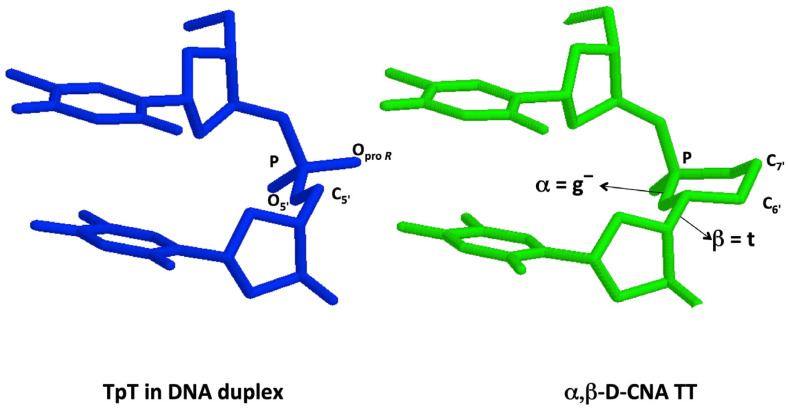
Initial hypothesis for the sugar/phosphate backbone conformational control by construction of a 1,3,2-dioxaphosphorinane moiety.

Therefore, with the aim of developing a rational approach to nucleic acid conformational control, we sought to have a chemical modification able to constrain one or a set of the torsional angles defining the sugar/phosphate backbone. An initial observation based on the X-ray structure of a TT dinucleotide involved in a B-DNA duplex, showed that the 5′-carbon, the 5′-oxygen, the phosphorus, and one oxygen of the phosphate group, were in a relative geometry identical to the one they could have if they were involved within a six-membered ring in its thermodynamically stable chair conformation. This led us to propose that an ethylene connection (C_6_’-C_7_’) between one oxygen atom (O_proR_) of the phosphate group and the 5’-carbon of the sugar ring could provide a conformational lock of the B-conformation of this dinucleotide step (Figure 5).

Consequently, the torsional angles α and β would be constrained by the newly created six-membered ring 1,3,2-dioxaphosphorinane, into the B-type duplex canonical values gauche(−) and trans, respectively. Therefore, we hypothesized that the so-called α,β-D-CNA (dioxaphosphorinane-constrained nucleic acid), because they represent a rigid pre-organized structure of the dinucleotide step observed in the B-type duplex, should be able to induce an interesting effect on duplex formation ability. A corollary of this hypothesis derived from the fact that this modification created two new asymmetric centers, one at the 5′-carbon, and the other at the phosphorus atom, offering the possibility of four diastereomers. Of course, only one fits with the geometrical requirements of a B-type duplex, but it opens up the opportunity to have in hand other fixed sugar/phosphate backbone geometry that could be of interest to mimic or stabilize disparate nucleic acid conformations.

We first synthetized, by a diastereoselective methodology, the α,β-D-CNA diastereomers, and their structural characterizations fully matched with our initial hypothesis. Lately, we have completed the alpha torsional angle set value by synthesizing a CNA, denoted as P-CNA, in which the dioxaphosphorinane ring is replaced by a phostone (six- membered cyclic phosphonate) through an intramolecular Arbuzhov reaction. This approach has allowed us to prepare all the possible α,β-D-CNA diastereoisomers [123]. Finally, the family was completed by the thio- and seleno-α,β-D-CNA diastereoisomers that displayed the same torsional angles values as their oxo-counterparts [124].

**Figure 6:**
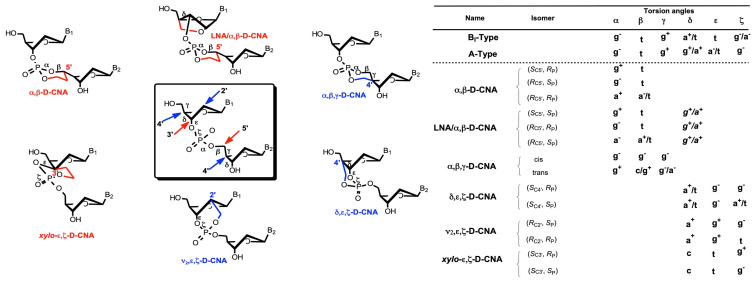
D-CNA family and determined torsional angles.

For the purpose of expanding the set of covalently constrained nucleic acids (CNA) with specific canonical or non-canonical backbone conformations, we have extended the approach proposed for α,β-D-CNA to the control of five other sets of torsional angles [125,126,127,128,129,130,131,132]. Firstly, we connected the phosphate, either to the 4′-C carbon of the lower sugar moiety to generate α,β-D-CNA, or to the upper sugar to obtain δ,ε,ζ-D-CNA. Starting from the sugar pool, we prepared *xylo*,-ε,ζ and ν_2_,ε,ζ-D-CNA. Eventually, double-constrained dinucleotides, combining a LNA and an α,β-D-CNA, were synthetized showing the versatility of the synthetic method in which a canonical sugar-puckering (locked North conformation) can be associated either with a canonical or not α,β conformation. All these units have been structurally characterized and the values of the corresponding constrained torsional angles are reported in Figure 6.

**Figure 7:**
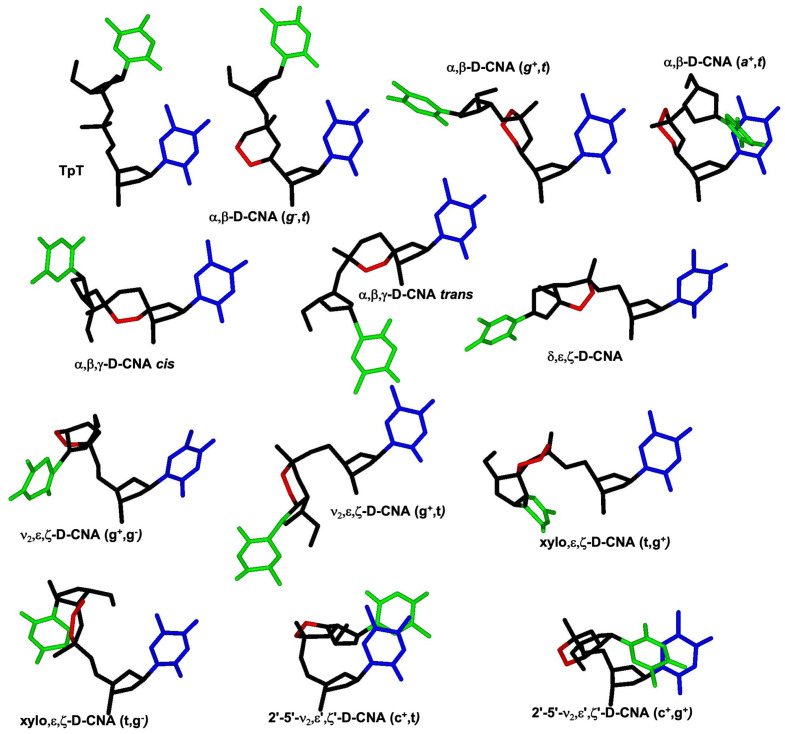
Representation of X-ray diffraction or NMR-based structures of selected CNAs.

To emphasize the topological diversity exhibited by these D-CNA structures, we have collected all the pictures extracted from X-ray diffraction, NMR structural studies, and molecular simulations. The di-thymidilate phosphate (TpT) X-ray structure is shown as a reference and all the CNAs are represented by positioning the lower thymidine (blue) in the same manner in order to better visualize the relative position of the upper thymidine (green) in these dinucleotide units (Figure 7).

It can clearly be seen that the topological landscape covered by the CNA dinucleotide units is wide and represents a very interesting toolbox for the fine-tuning of local and disparate DNA structures.

### 3.2. Behavior of α,β-D-CNA within Oligonucleotides

**Figure 8:**
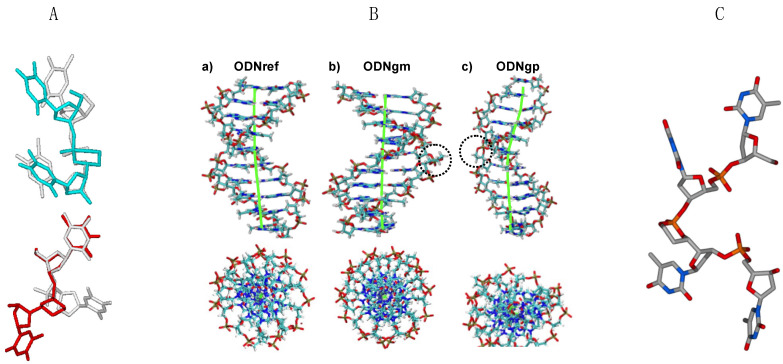
α,β-D-CNA within ON. (**A**) Superimposition of (g^−^, t) blue and (g^+^, t) red α,β-D-CNA with unmodified TpT. (**B**) Molecular dynamics simulation of unmodified dA_10_/dT_10_ (ODNref), of α,β-D-CNA, featuring canonical torsional angle value within dA_10_/dT_10_ (ODNgm), and of α,β-D-CNA featuring non-canonical torsional angle value within dA_10_/dT_10_ (ODNgp). (**C**) α,β-D-CNA (g^+^, t) within tetrathymidilate.

As a proof of concept, we showed that when reducing the conformational states of DNA single strands to those that match the geometry of the strand in duplex form, by locking α and β torsional angles within α,β-D-CNA (g^−^, t), duplex stabilities were increased by +5 °C/mod and +3 °C/mod towards their DNA and RNA counterparts, respectively (Figure 8), and the selectivity was preserved towards mismatched base-pairing [133,134,135]. In α,β-D-CNA, the sugar-puckering of the upper nucleotide is pushed into the C2’-endo conformation by the loss of the phosphate charge whatever is the nature of the sugar: 2’-deoxyribose, ribose, or 2’-*O*-methylribose [136]. It is noteworthy that the stabilization effect of α,β-D-CNA (g^−^, t) on duplex formation decreases with the length of the oligomer, as expected, and it is strictly additive when placing two or three modifications on the same strand or one on each complementary strand.

On the other hand, and very interestingly, the α,β-D-CNA (*S*_C_, *R*_P_) dinucleotide featuring unusual torsional angle values (g^+^, t) still formed a duplex but with a local distortion observed only in the DNA/protein complex. These duplexes are still base-paired, as indicated by mismatching studies, but, of course, with a loss in thermal stability (−9 to −6 °C/mod depending on the length and composition of the oligomer). Therefore, this is a rare example in which thermal destabilization can be correlated with torsional stress within a duplex.

The two major isomers of α,β-D-CNA, i.e., (*R*_C5’_, *S*_P_) with the conformation (g^−^, t), and (*S*_C5’_, *R*_P_) with the conformation (g^+^, t), nicely illustrate the concept of pre-organization for nucleic acid secondary structure stabilization. By locking a TT step into the B-type canonical conformation, we observed that rigidity was induced within the DNA duplex, whereas introduction of a torsional stress (alpha from g^−^ to g^+^) either resulted in a slight bend in the duplex structure or in a preorganized single strand suitable for hairpin loop structure stabilization (Figure 8C) [137].

This latter observation led us to investigate the opportunity for the incorporation of the α,β-D-CNA (g^+^, t) within the impaired moiety of hairpin or bulge structures in accordance with the preorganization concept, to provide for the first time stabilization of nucleic acid secondary structure by control of the sugar/phosphate backbone.

**Figure 9:**
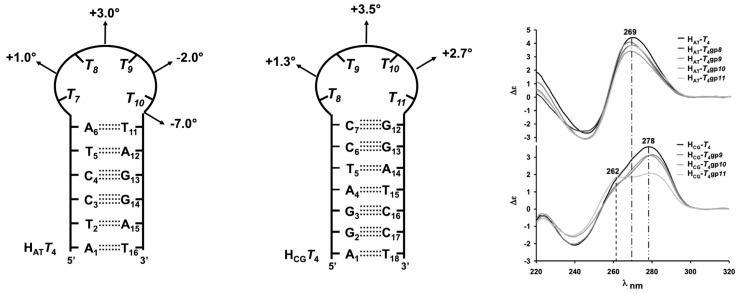
α,β-D-CNA (g^+^, t) within hairpin structures.

We studied two kinds of hairpins where loops are composed of four or five thymidines with closing base pairs made of AT (H_AT_T_4/5_) or CG (H_CG_T_4/5_). On these structures all the TT steps were replaced by an α,β-D-CNA TT (g^+^, t) one-by-one and the corresponding thermal stability was evaluated (Figure 9) [138].

On H_AT_T_4_ there are four possible positions for the incorporation of the alpha g^+^ constraint, and it turned out that two of them were favorable, with a maximum of +3 °C in thermal stabilization when located in the middle of the loop (between T_8_ and T_9_). However, this constraint is not tolerated with a strong loop in thermal stability (−7 °C) if it is applied at the connection between the loop and the stem but also to a lower extent at the T_10_-T_11_ position. The recorded CD spectra did not show a change in the overall shape when compared with the unmodified hairpin and exhibited all of the characteristics of a B-type duplex.

With a CG closing base pair, the impact of the α,β-D-CNA TT (g^+^, t) was positive, wherever it was installed in H_CG_T_4_, and slightly more important than for H_AT_T_4_ (+3.5 °C). However, it was very surprising that in H_CG_T_4_, the alpha g^+^ constraint was stabilizing at the 3’-end of the loop whereas it was detrimental for H_AT_T_4_. A potential explanation came from the recording of the corresponding CD spectra that showed a change in the shape with a new maximum in the positive Cotton band at 262 nm, whereas the unmodified hairpin exhibited only one at 278 nm. This observation could be indicative of a partial switch of the stem to A-form helices; therefore this stem rearrangement could explain the gain in thermal stability. Eventually, we showed that with an alpha g^+^ constraint applied in the loop, it was possible to reduce the length of the stem down to two base pairs and still have a hairpin structure, either by a preorganization effect or by a conformational change of the stem.

The same behavior of α,β-D-CNA TT (g^+^, t) was shown for H_AT_T_5_ and H_CG_T_5_ but with induction of an extended hairpin thermal stability of +4 and +5 °C, respectively.

Similar to the hairpin loop structure, nucleic acid bulges exhibit sharp changes in the sugar/phosphate backbone in both strands, but to a lower extent, since the directionality of the looped strand is not completely reversed. The impaired bases forming the bulge induce a kink of the two stems in the overall structure, generally ensured by one phosphate in the bulge moiety, and by the phosphate connecting the stems.

**Figure 10:**
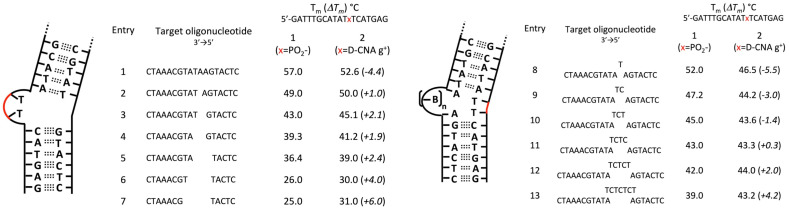
α,β-D-CNA (g^+^, t) within bulge structures. (**Left**): within the bulged moiety. (**Right**): opposite to the bulged moiety.

Therefore, we examined the potential for a preorganization effect of α,β-D-CNA TT (g^+^, t), when installed either within or opposite to the bulged moiety, on the stability of bulge structures with a size loop varying from one to six nucleotides (Figure 10).

The fully matched duplex is destabilized by −4.4 °C by one incorporation of α,β-D-CNA TT (g^+^, t) and as soon as an one nucleotide bulge structure was created, the alpha g^+^ constraint proved to be a stabilizing element. From a two to four nucleotide loop the stabilization was modest (+2 °C) but increased significantly for five and six nucleotide bulged structures, reaching +4 and +6 °C, respectively.

The kink degree between the two stems induced by the bulge is proportional to its number of nucleotides. This feature can explain the behavior of the alpha g^+^ constraint when located opposite to the bulge. Whatever the bulge size was, it was noticeable that all the bulge structures, from two to six nucleotides in size, were characterized by roughly the same melting temperature (44 ± 1 °C), with the exception of a single unpaired base that was slightly more stable. But when compared with the unconstrained counterparts, the stabilizing effect of the alpha g^+^ constraint only arose when four bases were excluded (+0.3 °C), and became efficient with five and six base bulges with +2 and +4.2 °C, respectively. It can be postulated that the kink of the flanking helices is better mimicked by the constraint deviating by 120° from the canonical value with the α,β-D-CNA TT (g^+^, t) for bulges larger than four nucleotides.

These were the first examples of extended stabilization of hairpin or unstable bulged structures by sugar/phosphate constraints applied to the unpaired nucleotides moiety with respect to the sequence composition.

This work was completed by a thorough study to decipher the impact of the g+ constraint, either in a larger loop while varying the nature of the AT or CG loop-closing base pair, or when introduced in or opposite to bulges of different sizes [139].

Therefore, the D-CNA approach to introduce conformational restriction within nucleic acids is the only one with a rationale based on a perfect knowledge of the torsional angle constrains.

### 3.3. In Vitro Properties of α,β-D-CNA

#### 3.3.1. α,β-D-CNA Used as Terminators of Polymerase Chain Reaction

The particular ability of α,β-D-CNA (g^+^,t) to promote a marked bending within oligonucleotides led us to propose to use its particular behavior to generate a DNA template able to produce a terminator structure of polymerization in the polymerase chain reaction (PCR). These terminators are of particular interest in the systematic evolution of ligands by exponential enrichment for DNA aptamer selection by the production of two DNA strands of different length to enable their easy separation and purification.

**Figure 11:**
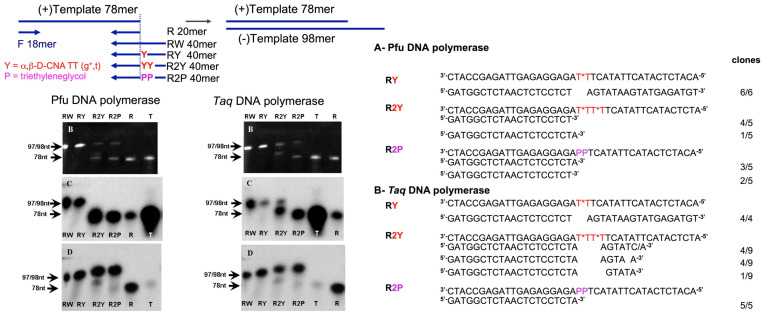
Left: primer design with one or two α,β-d-CNA units (Y) or triethylene glycol unit (P). Gel electrophoresis analysis of PCR products with proof-reading polymerase Pfu, or without proof-reading polymerase *Taq*. Right: sequencing of terminated PCR products.

We therefore assessed the efficiency of α,β-D-CNA as a terminator in PCR, using triethylene glycol phosphate units as controls; one or two α,β-D-CNA units were incorporated into primer PCR products and were further analyzed either by gel electrophoresis or sequencing (Figure 11) [140].

Both polymerases skipped one incorporation of α,β-D-CNA within the primer (RY), producing a transcript where two nucleotides were missing, as attested by clone sequencing. On the other hand, proof-reading polymerases, such as *Pfu* DNA polymerase, were stopped by incorporation of two consecutive constrained units (R2Y) and can therefore be classified as strong replication terminators, similar to consecutive triethyleneglycol phosphate moieties (PP). A non-proof-reading enzyme, e.g., *Taq* DNA polymerase, was not so sensitive to the α,β-D-CNA structure as a strong terminator, though it was eventually stopped but only after elongation of several more nucleotides.

This study lead us to propose that CNA and their derivatives could be useful tools to investigate the behavior of different classes of polymerases.

#### 3.3.2. α,β-D-CNA Used for Allele Selective Silencing

RNase H-mediated activity and the allele selectivity of antisense oligonucleotides (ASO), modified by α,β-D-CNA featuring either the canonical constraint (g^−^,t) or the non-canonical (g^+^, t), in the context of single nucleotide polymorphism targeting (SNP), have been evaluated for the treatment of Huntington’s disease.

**Figure 12:**
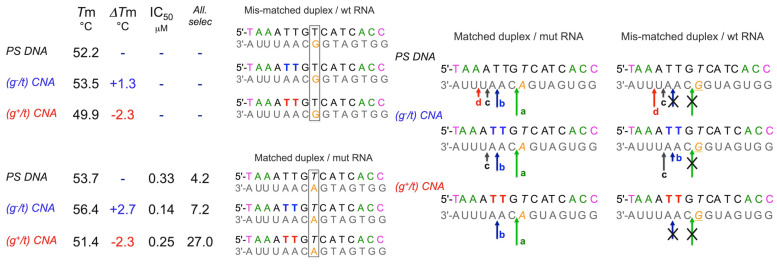
Left: Sequence, chemical modification pattern, thermal melting temperature (Tm) vs wild type RNA with a mismatch (wt RNA) or mutant RNA fully matched (mut RNA), activity against mutated Huntington protein (IC_50_) and fold selectivity vs wild type Huntington protein (All. Selec) in human fibroblasts. Right: Cleavage pattern by recombinant human RNase H of heteroduplex in a biochemical assay. All ASOs are phosphorothioate wherever possible. Magenta letters denoted 2’-*O*-methoxyethyl RNA (MOE), green denoted 2’-*O*-ethyl BNA, blue denoted α,β-D-CNA (g^−^,t), and red α,β-D-CNA (g^+^,t). Black denoted 2’-deoxynucleotides and grey ribonucleotides.

ASOs were built with 2’-deoxynucleotides or ribonucleotides with phosphorothioate linkage and flanked by 2’-*O*-methoxyethylRNA and 2’-*O*-ethylBNA to ensure nuclease stability and to enhance duplex stability by reducing end frying. In this a so-defined gapmer (9-nucleotides long) was introduced with one CNA two nucleotides away from the spot mutation (Figure 12) [141].

As expected, duplex thermal stability compared to complementary and mismatched RNA was increased by the canonical constraint, and the non-canonical constraint induced the same loss of −2.3 °C independently of the matched or mismatched target. Interestingly, the RNase H activity profile was similar for (g^−^,t)-CNA and the reference ASO suggesting that this modification can support the RNase H activity in its vicinity. However, the allele selectivity was poorly improved because only the major cleavage site (site a, Figure 12) has been ablated. On the other hand, while the (g^+^, t)-CNA acted roughly as its counterparts towards the matched duplex, it completely ablated the cleavage site towards the mis-matched duplex inducing a high allele selectivity. Therefore, these results suggested that the efficiency of nucleic acid modification should not only be highlighted for its duplex formation ability, especially when extra enzyme activity is necessary with a local geometry flexibility requirement for its action. Subtle local structural distortion can therefore generate interesting biological properties and these relationships in a system of interest can allow for the determination of the true impact of oligonucleotide chemical modification for the purpose of antisense applications.

## 4. Structuration and Functionalization of Nucleic Acids (C_2_NA)

Finally, some of the last developments concerned the combination of both labeling and conformational restriction to confer complementary and beneficial properties in the development of therapeutic oligonucleotides, or for diagnostic applications.

Most of the efforts were initiated by the Wengel laboratory, based on the 2’-modified-2-amino-LNA scaffold, by conjugating the resulting constrained ON with cationic (poly)amines to increase its stability towards nucleases and its cellular uptake by shielding its anionic nature.

For gene-silencing applications, they synthetized already functionalized LNA monomers conjugated with a glycyl moiety further incorporated into a triplex forming oligonucleotide, and their presence was proven to be stabilizing [142]. Following the same synthetic strategy, they also introduced a positively charged piperazino group [143] or diamino groups [144] to increase binding affinity and resistance to 3’-exonucleotlytic degradation. Finally, they also relied on a convertible approach with the 2’-alkynyl-2-amino-LNA phosphoramidites, allowing up to two post synthetic modifications by different polyamines [145].

**Scheme 9:**
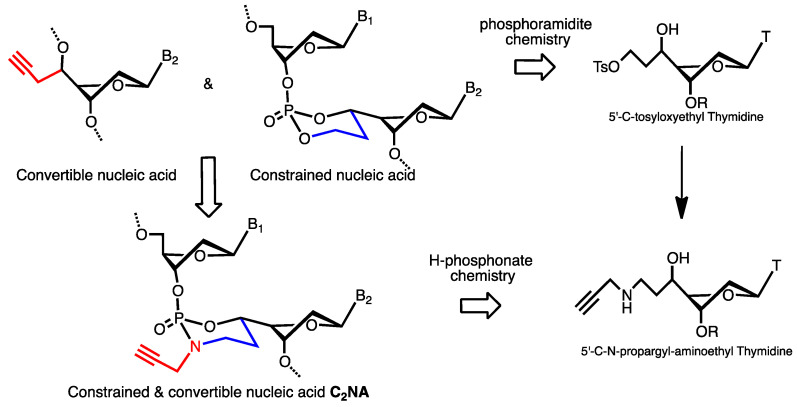
Conceptual merging of the convertible function and the conformational constraint to provide the double property of constrained and convertible nucleic acid (C_2_NA).

Eventually, the two research programs that we were engaged in were merged to combine in one dinucleotide structure properties brought together by both the convertible and the constrained approach. As a consequence, it became obvious that the appended propargyl arm should be connected to the phosphorinane moiety. We designed an *N*-propargyl-1,3,2-oxaza-phosphorinane as an internucleotidic linkage to fulfill all the requirements of this class of compounds denoted as convertible and conformationally constrained nucleic acids (C_2_NAs, Scheme 9) [146].

While the synthesis of CNAs was based on the phosphoramidites technology, we turned to the *H*-phosphonate chemistry to obtain the C_2_NAs, by a different procedure, from the key intermediate 5’-C-tosyloxyethyl thymidines that became the common precursors for the two families (Scheme 9).

**Figure 13:**
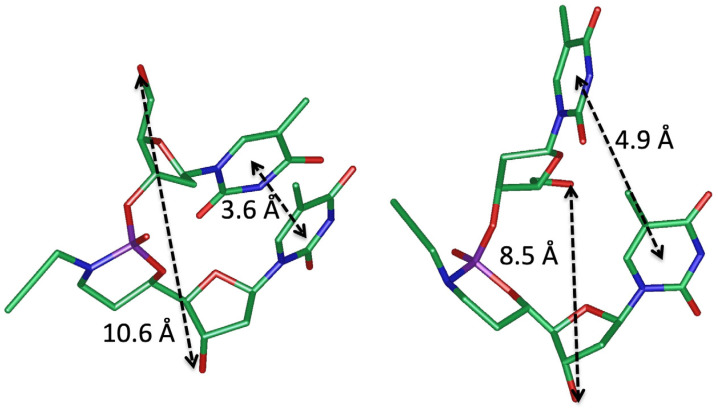
Lowest energy conformations from the in silico protocols. Left: (*R*_C5’_, *R*_P_)-α,β-C_2_NA. Right: (*S*_C5’_, *S*_P_)-α,β-D-C_2_NA.

We prepared two diastereoisomeric 5’-C-*N*-propargyl-aminoethyl thymidines that were condensed with the thymidine *H*-phosphonate derivative to generate two major 5’-C “*S*” and 5’-C “*R*” α,β-C_2_NAs isomers. Identically to their α,β-D-CNAs counterparts, the major α,β-C_2_NAs isomers featured a chair conformation for the oxaza-phosphorinane rings as revealed by NMR study and in silico conformational calculations. Therefore, the constraints of the torsional angles α and β were determined to be similar to those observed for the corresponding D-CNA, the (*R*_C5’_, *R*_P_)-α,β-C_2_NA featured the *gauche*(−), *trans* configuration and, in contrast, the (*S*_C5’_, *S*_P_)-α,β-C_2_NA differed with the *gauche*(+), *trans* values from the canonical set of torsional angle values. Interestingly, in silico calculation results showed that the propargyl function appended to the oxaza-phosphorinane ring was totally accessible (Figure 13) for a further functionalization through the copper-catalyzed Huisgen cycloaddition (CuAAC click chemistry).

In line with the NMR study, in silico calculations confirmed the relative positioning of the thymine bases that were prone to stack within the (*R*_C5’_, *R*_P_))-α,β-C_2_NA isomer when they located in two nearly perpendicular arrangements for the (*S*_C5’_, *S*_P_)-α,β-C_2_NA isomer. As a consequence, the 5’ and 3’ hydroxyl functions were separated by approximately 11 Å for the isomer featuring the canonical values of the torsional angles, and were closer by 2 Å when α deviated by 120° from the canonical *gauche*(−) value. As expected, the (*R*_C5’_, *R*_P_)-α,β-C_2_NA isomer fitted with a dinucleotide involved in a B-type duplex when the (*S*_C5’_, *S*_P_)-α,β-C_2_NA isomer didn’t and was expected to be better suited to the unpaired structure of nucleic acids.

From these results it can also be postulated that, once incorporated within an oligonucleotide and annealed to form a duplex, the appended arm would be oriented out of the double helix together with the newly grafted chemical functionality or biologically relevant molecule.

**Figure 14:**
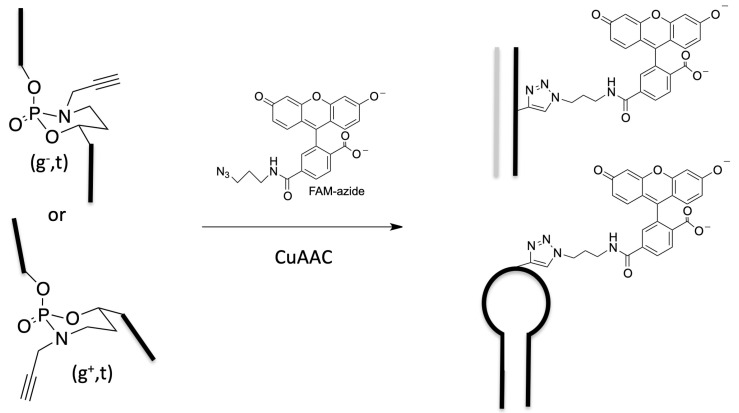
α,β-C_2_NA modified ONs conjugation with fluorescein for duplex or hairpin decoration.

We have investigated the behavior of the α,β-C_2_NA modification in both conformations within the duplex and within the hairpin and bulge for the non-canonical isomer, before and after coupling with fluorescein by a classical and efficient CuAAC reaction used as proof of concept for the convertible and conformational approach (Figure 14).

The (*R*_C5’_, *R*_P_)-α,β-C_2_NA featuring the *gauche*(−), *trans* configuration was a stabilizing element of the duplex with an average increase of the Tm value of +3 °C, which was less than the corresponding value for α,β−CNA. Once the fluorescein attached, the melting temperature decreased, but it appeared that the impact was sequence-dependent and that the negative effect of the dye grafting upon duplex stability could be partially or totally compensated by the canonical constraint.

On the other hand, the duplex including the (*S*_C5’_, *S*_P_)-α,β-C_2_NA featuring the *gauche*(+), *trans* configuration was strongly destabilized by −9 °C, identical to what was observed with the α,β-CNA analogue. The duplex destabilization was increased by the covalent grafting of the hydrophobic fluorescein moiety, reaching −11 °C when compared to the wild type.

The α,β-C_2_NA *gauche*(+), *trans* dinucleotide incorporated within the loop of a bulged structure was less effective towards modification of the thermal stability. However, in opposition to the growing loop, this modification was to a degree effective since the loop size reached four nucleotides with an average stabilization of +2 °C of the bulge and was therefore comparable with the α,β−CNA featuring the *gauche*(+), *trans* conformation.

In contrast, when installed within the unpaired moiety of a four nucleotide looped hairpin, the (*S*_C5’_, *S*_P_)-α,β-C_2_NA was able to induce the strongest stabilization level observed to date of +6 °C. Interestingly, after decoration with fluorescein, the thermal stability of the hairpin decreased compared with the uncoupled form (−4 °C), but stayed higher by +2 °C with respect to the unmodified version.

The α,β-C_2_NAs fulfill all the requirements to become very interesting tools that could be used in many applications whether for biological investigations such as exon skipping, gene interference, or antisense therapeutic applications, or for biotechnological development, since they combine good stability, target affinity modulation, and the opportunity to be decorated by any “clickable” molecules.

## 5. Conclusions

Functionalization, decoration, or conformational control of nucleic acids is still a major challenge and a field of intense research due to the tremendous need for therapeutic, biological, or biotechnological applications of chemically modified oligonucleotides. Among the many potential positions for derivativization along the oligonucleotide chain, we focused on the 5’-carbon of the sugar residue and showed that it was a location of choice to prepare convertible nucleotides (CvN) since it allows a straightforward conjugation at any point of the chain without disturbing the pairing properties. Moreover, the chemistry developed around the 5’-C functionalization opens the way to the constrained nucleic acid (CNA) building blocks that exhibit very interesting properties for the conformational control of oligonucleotides, either by stabilizing duplex structures or the unpaired moieties of hairpins or bulges. Ultimately, the combination of both approaches lead to the C_2_NAs (constrained and convertible nucleic acids) that are promising tools exhibiting the two properties. These molecules have been developed in DNA series but there is no restriction to applying these methodologies to the RNA series and we are currently working on this.

In sum, the above mentioned CvNs and CNAs can be used for the concept and development of biologically active oligonucleotides in exon skipping or antisense therapy. For these applications, these new agents will need to have increased stability and affinity for the targeted sequence, in addition to the ability to be conjugated with specific molecules to reach a specific biodistribution. This toolbox could be used in biotechnological applications, such as single nucleotide polymorphism detection, DNA nanostructures, and origami decoration by site and regio-specific appended new chemical functionalities, or even in the more challenging targeting of highly biorelevant non-canonical structures displaying roles in cancers or neurodegenerative diseases [147,148].

## Data Availability

Not applicable.

## Sample Availability

Samples of the compounds are not available from the authors.
